# Exosomal microRNAs in hepatocellular carcinoma

**DOI:** 10.1186/s12935-021-01941-9

**Published:** 2021-05-08

**Authors:** Chenbin Liu, Han Wu, Yinqi Mao, Wei Chen, Shuying Chen

**Affiliations:** 1 School of Medicine, Shanghai Jiao Tong University, 227 Chongqing South Road, Shanghai, 200025 China; 2 Department of Laboratory Medicine, Huashan Hospital, Fudan University, 12 Wulumuqi Middle Road, Shanghai, 200040 China

**Keywords:** Hepatocellular carcinoma, Exosomes, microRNA, Biomarkers

## Abstract

Hepatocellular carcinoma is one of the most common malignant tumors worldwide and the fourth leading cause of cancer-related deaths. The prognosis of hepatocellular carcinoma patients is extremely poor due to the occult onset and high metastasis of hepatocellular carcinoma. Therefore, biomarkers with high specificity and sensitivity are of great importance in early screening, diagnosis prognosis, and treatment of hepatocellular carcinoma patients. Exosomes are tiny vesicles secreted by various types of cells, which can serve as mediators of intercellular communication to regulate the tumor microenvironment, and play a key role in the occurrence, development, prognosis, monitor and treatment of hepatocellular carcinoma. As microRNA deliverer, exosomes are involved in multiple life activities by regulating target genes of recipient cells such as proliferation, invasion, metastasis and apoptosis of cancer cells. In this review, we summarized the composition, active mechanism and function of exosomal microRNAs in hepatocellular carcinoma, and elaborated on their potential application value of early diagnosis and treatment in hepatocellular carcinoma.

## Introduction

Hepatocellular carcinoma (HCC), the main form of primary liver cancer, is the sixth most common malignant tumor in the world and the fourth leading cause of cancer-related deaths globally [1, 2]. Due to its occult onset, the high degree of malignancy and poor prognosis, HCC is a serious threat to human’s physical and mental health HCC is considered the major cause of death in patients with cirrhosis, and its morbidity is estimated to increase in the future [3, 4]. Most hepatocyte cases (80 %) occur in sub-Saharan Africa and East Asia, where the major risk factors are chronic hepatitis B infection and exposure to aflatoxin B1 [5].

The development of HCC is a complex multi-step process including persistent inflammatory damage and hepatocyte necrosis and regeneration associated with fiber deposition. The occurrence of HCC is the result of both epigenetic modification and the accumulation of somatic genomes mutation, which explains its great molecular heterogeneity [6]. Seeking reliable biomarkers for screening and diagnosis of hepatocellular carcinoma has important clinical application value [7]. Serum tumor markers are effective for HCC surveillance and early diagnosis because of the less-invasive, objective, and repeatable assessment. Alpha-fetoprotein the most commonly used serologic test item, but in retrospective case-control studies, even with the most effective threshold (10–20 ng/mL), the reported sensitivity was around 60 % and specificity was only 80 % [8, 9].The main treatment options for HCC are surgical resection, transplantation, ablation, transarterial chemoembolization [10–12], and drug therapy such as sorafenib [13, 14], levatinib [15], and regorafenib [16]. They are all tyrosine kinase inhibitors. In recent years, extensive studies on the biochemical properties and biological functions of exosomes have been conducted, and both in vitro and in vivo experiments have confirmed that exosomes may play a vital role in the occurrence, development, diagnosis and treatment of HCC, which can provide new insights for a comprehensive understanding of HCC [17].

Exosomes are nanovesicles with a diameter of 50–150 nm secreted from different cell types into the extracellular environment by fusion with the cell membrane [18]. They contain proteins and various genetic materials, including mRNA, microRNA, and other ncRNAs [19]. They are involved in intercellular communication and microenvironment regulation by mediating signal pathways in recipient cells and thus play a crucial role in chemical resistance, angiogenesis [20], epithelial-mesenchymal transition (EMT) [21] and metastasis of tumors [22]. On the one hand, tumor cells affect neighbor cells through exosomes and establish a tumorigenic microenvironment. On the other hand, matrix cells (such as stellate cells and mesenchymal stem cells) and immune cells may interfere with tumor cells to promote or prevent tumorigenesis through exosomes [23].

MicroRNAs are a class of conservative small non-coding RNAs of 17-24nt in length. By complementary base pairing with target mRNA, they lead RNA-induced silencing complex (RISC) to degrade mRNA or cut off its translation, inhibiting protein expression, and thus involved in post-transcriptional control [24, 25]. MicroRNA proved to be involved in many biological activities, including cell proliferation, cell differentiation, cell migration, disease initiation and progression [26–29]. Lotvall et al. first proposed the “exosome shuttle RNA (esRNA)” hypothesis in 2007, confirming that exosomes from mouse mast cells contain mRNAs and microRNAs which can be delivered into human mast cells and function in recipient cells [30]. The imbalance of exosomal microRNA expression can accelerate disease progression and affect the pathophysiological status of tumor and its abnormal expression is closely related to the occurrence and development of tumor [31, 32]. The latest studies found that microRNAs mediated by exosomes also play a crucial role in the occurrence and development of liver cancer [33]. Considering that liver biopsy is the gold standard for monitoring and evaluating liver disease but with a risk of bleeding and infection, less-invasive diagnostic tools are urgently needed [34]. Therefore, the detection of serum exosomal microRNA for early diagnosis and prognosis prediction of HCC becomes attractive. In addition, compared with cell therapy, cell-free exosome therapy has lower immunogenicity with stable contents and is easy to store and mass produce, preventing many risks and difficulties. Cell-free exosome therapy represents a promising new treatment method [35, 36].

The previously published reviews mostly focused on the related functional mechanisms of exosomes in HCC[17, 37–39]. In this review, we summarized the expression changes and the functions of exosomal microRNAs in HCC. We also discussed the changes of exosomal microRNA/signaling pathway. Finally, we further discussed the clinical application values of exosomal microRNAs as biomarkers for HCC.

## Changes in the expression of microRNA in HCC exosomes

More and more studies have shown that the expression of exosomal microRNAs is closely related to the progression of HCC, and can even be used to distinguish tumors of different grades. As early as 2014, Wang et al. detected the expression of exo-miR-21 in serums of patients with HCC or chronic hepatitis B, and evaluated its potential for early detection of HCC. Researchers found that the expression level of exo-miR-21 in patients with HCC was significantly higher than that in patients with chronic hepatitis B or healthy persons, and had a higher diagnostic sensitivity [40]. Another research used microRNA deep sequencing to screen differentially expressed microRNAs in HCC and cirrhosis, and then utilized qPCR to verify candidate microRNAs in an independent verification cohort. They found that the expression of exo-miR-122, exo-miR-148a and exo-miR-1246 in serum of HCC was significantly higher than that of liver cirrhosis and normal control group, and the combination of exosomal microRNA and alpha-fetoprotein (AFP) produces better diagnostic performance than AFP in distinguishing the receiver operating curve of HCC and liver cirrhosis. It suggested that exosomal microRNAs or their combination with traditional biomarkers may become promising markers for the screening and diagnosis of HCC[41]. In addition, in the study of Lin et al.[42], the significantly increased levels of 19 microRNAs in serum of HCC patients were first identified, and then miR-210 was found to be secreted by HCC cells via exosomes. In the study of Sohn et al.[43], serum exosome microRNAs were extracted from the serum using the exosome RNA isolation kit, and microRNA expression levels were quantified by real-time PCR. The serum levels of miR-18a, miR-221, miR-222 and miR-224 in exosomes of HCC patients were significantly higher than those in chronic hepatitis B patients. The increase or decrease of exosomal microRNAs may reflect the progression of HCC or be related to the pathophysiology of liver cancer cells. In addition, because microRNAs are encapsulated by exosomes, they have high stability and are not easily affected by the external environment, making them of high application value in the clinical diagnosis of HCC. Therefore, with the in-depth study of the functions of differential microRNAs in these exosomes and the understanding of valuable information about exosomes-related HCC, the application value of differentially expressed exosomal microRNAs will be further determined, in order to establish an strong foundation for being potential diagnosis and prognostic markers of HCC.

## The changes of exosomal microRNA/signaling pathway

Exosomal microRNA participates in the pathological process of multiple tumors, including HCC, playing an extremely important role in the environmental regulation of HCC generation and development. In the past few years, more and more articles have suggested that there are many dysfunctions of signal pathways in the generation and development of primary hepatic carcinoma (PHC). However, the regulation effect and mechanism of these signal pathways involved in PHC occurring and developing have not been completely clarified, remaining to be further discussed. The following content is a review of the influence of exosomal microRNAs in the occurrence of PHC and of some essential relevant molecular pathways and mechanisms. Exosomal microRNA is regarded as a promising biomarker for diagnosis, prediction, prognosis, and real-time monitoring of the therapeutic reaction of PHC. A further understanding of exosomes in HCC will bring a breakthrough change in the diagnosis and treatment of HCC in the future.

### Phosphatidylinositide 3-kinase (PI3K)/serine/threonine kinase (akt) signaling

PI3K catalyzes the formation of phosphatidylinositol-4-phosphate and phosphatidylinositol-4,5-bisphosphate(PIP2) [44]. Growth factor and hormone may trigger the event of phosphorylation, which in turn regulates cell growth, cell cycle entry, cell migration, and cell survival [45, 46]. The downstream signaling of PI3K is mediated through Akt, which plays a key role in the regulation of cell apoptosis by pro-apoptotic proteins such as caspase-9 and Bad [47]. Akt, also known as PKB or Rac, is essential for controlling survival and apoptosis [48–50]. This protein kinase is activated by insulin and a variety of growth and survival factors, and can inhibit apoptosis, thereby promoting cell survival [51]. In addition to its role in cell survival and glycogen synthesis, Akt is involved in cell cycle regulation by negatively regulating the cyclin-dependent kinase inhibitors p27 Kip1 [52] and p21 Waf1/Cip1 [53]. The PI3K/Akt pathway is considered to be a molecular “crutch” for cells to escape death [54], and contributes to progression in different types of cancers by regulating proliferation, apoptosis, angiogenesis, EMT, and autophagy [55, 56]. A large number of research indicates that microRNA plays an important role in the occurrence, development, metastasis and treatment of HCC through the phosphatase and tensin homolog(PTEN)/PI3K/Akt pathway [57]. In the study of Xiao Fu et al. exo-miR-32-5p is transmitted from drug-resistant cells to sensitive cells through exosomes [58]. It activates the PI3K/Akt pathway and induces multidrug resistance by regulating angiogenesis and EMT. Another research shows that the exo-miR-23a released by endoplasmic reticulum stressed HCC cells regulates Programmed Cell Death-Ligand 1 (PD-L1) expression and inhibits T cell function through PTEN-Akt pathway [59]. PI3K/AKT pathway is one of the most important signal transduction pathways related to the control and growth of HCC [60]. Nowadays, the development of PI3K inhibitors for the treatment of cancer has been limited by the dose and targets the challenge of adverse reactions [61]. As a result, continuing to perfect the views on how various PI3K enzymes work in HCC will provide new opportunities for therapeutic intervention in HCC and other diseases.

### **Mitogen-activated protein kinases (MAPKs)*/extracellular regulated protein kinases (ERK) in growth and differentiation***

MAPKs are a family of widely conserved serine/threonine protein kinases involved in multiple cellular processes, such as cell proliferation, differentiation, motility and death [62]. P44 / 42 MAPK (ERK1 / 2), mitogen-activated protein kinase 9 (SAPK)/c-Jun N-terminal kinase (JNK) and p38 MAPK play a key role in protein kinase cascade, and protein kinase cascade plays a significant role in regulating cell growth and differentiation and controlling cell response to cytokines and stress [63]. P44/42 MAPK (ERK1/2) signal transduction pathway can be activated in response to a variety of extracellular stimuli (including mitogens, growth factors and cytokines) [64–66], and it is an important target for cancer diagnosis and treatment [67]. Some studies have shown that exo-miR-320a suppress HCC cell proliferation, migration and metastasis by inhibiting the activation of MAPK pathway, which can induce EMT and upregulate the expression of cyclin-dependent kinase 2 (CDK2) and matrix metallopeptidase 2 (MMP2) [68]. In other studies, overexpression of exo-miR-9-3p reduces the viability and proliferation of HCC cells, as well as the expression of ERK1/2 [69]. In vitro and in vivo, HCC cell-derived exo-miR-665 can promote the proliferation of hepatoma cells and upregulate the protein expression level of MAPK/ERK pathway [70]. In the study of Li Xiong et al., mast cells can inhibit the ERK1/2 pathway by transferring the exo-miR-490 into HCC cells, thereby inhibiting the metastasis of HCC cells [71]. Activation of the MAPK signaling pathway is a common event in tumor progression and metastasis [72]. These studies can provide new insights into the regulatory mechanism of HCC in the MAPK signaling pathway and identify potential ways of the therapeutic intervention for the disease.

### Inhibition of apoptosis

Apoptosis is a form of programmed cell death, which can remove damaged cells in an ordered and effective way, such as Cell with DNA damage or during development [73]. Apoptosis can be triggered by intracellular signals such as genotoxic stress, or by extracellular signals, such as the binding of the ligands to the cell surface death receptors [73]. One of the most important signals of cancer is the dysregulation of death mechanism in apoptotic cells [74]. Dysregulation in apoptosis not only relates to the occurrence and development of cancer, but also induces tumor resistance to the treatment [75]. Recently, some studies have shown that exosomal microRNA can regulate the occurrence and development of HCC via apoptosis. Studies have shown that exosomes from cancer stem cells in HCC can upregulate Bax (BCL2 associated X, apoptosis regulator), p53 and Bcl2 (BCL2 apoptosis regulator), thereby reducing tumor growth, progression and metastasis [76]. Researches by Jing Yang et al. proved that exo-miR-638 overexpression inhibits HCC proliferation by reducing viability, reducing colony formation, inducing apoptosis, leading cell cycle arrest in the G1 phase and reducing the ability of migration and invasion [77]. Exo-miR-451a, as a tumor suppressor, was also found to induce apoptosis both in HCC cell lines and human umbilical vein endothelial cells (HUVECs) [78]. Most anticancer drugs used in nowadays clinical oncology use apoptosis signaling pathway to trigger off the death of cancer cells [79]. Therefore, defects in the death pathway can lead to drug resistance, which can restrict the effectiveness of treatment [80]. A better understanding of exosomal microRNA regulation to apoptotic cell death signaling pathways may improve the efficacy of cancer treatment and bypass resistance [81].

## The role of exosomal microRNAs in the HCC microenvironment

### Tumor development and metastasis

Because of the high metastasis rate and high recurrence rate, the long-term survival rate of HCC patients is relatively low [82]. Tumor metastasis is a multi-step process, including invasion through the distal part of the circulatory system, vascular infiltration, and colonization [83]. Studies reveal that exosomes can create an immunosuppressive environment through signal transduction between stromal cells and tumor cells, thus affecting the progression of tumor [84]. Both exosomal microRNA from cancer cells and adjacent stromal cells can promote the formation of the metastatic niche [85–90]. Researches show that the loss of exo-miR-320a in the exosomes derived from cancer-associated fibroblasts (CAFs) in HCC can stimulate downstream ERK activation of receptor cells (hepatocytes), which results in lung metastasis [68]. Similarly, exo-miR-1247-3p in exosomes released by CAF can promote lung metastasis of HCC [91]. Other studies have found that macrophages can promote the invasion of hepatoma cells by secreting exosomes containing exo-miR-92a-2-5p [92]. Adipocytes can also secrete exo-miR-23a/b and transport them to cancer cells through exosomes, thereby promoting the growth and migration of HCC cells [93]. Exosomes released by cancer cells can also affect the proliferation and metastasis of tumors. Some researchers suggest that exo-miR-21 and exo-miR-10b in the exosomes of HCC induced by acidic microenvironment, can promote the propagation and metastasis of cancer cells. So they may be used as prognostic molecular markers and therapeutic targets of HCC [94]. These results indicate that microRNAs in exosomes can be transferred to target cells in the microenvironment of HCC, regulate the process of lung cancer cells, and construct a tumorigenic microenvironment, which leads to cancer metastasis.

### Angiogenesis

The growth and metastasis of tumors require new blood vessels to supplement oxygen and nutrients, so tumors can activate angiogenesis, allowing blood vessels to germinate from adjacent tissues into the tumor [95]. Angiogenesis is necessary for cancer growth and metastasis, and is regulated by a variety of molecules. Active angiogenesis is the cause of rapid tumor growth, early metastasis, and low survival rate [96]. Previous reports have shown that exo-miR-21, exo-miR-26a, exo-miR-122, exo-miR-146a, exo-miR-155 and exo-miR-182 are associated with HCC angiogenesis and prognosis [57, 97–102]. The exo-miR-210 secreted by HCC cells can be transferred to endothelial cells to promote tumor angiogenesis by targeting SMAD4 and STAT6 [42]. Other studies have shown that exo-miR-200b-3p from liver cells inhibit the expression of endothelial transcription factor ERG, and the reduction of exo-miR-200b-3p in cancer cells promotes the blood vessels of liver cancer tissues by enhancing the expression of endothelial ERG generate [103]. In conclusion, these results support exosomal microRNA as a novel pro-angiogenesis mechanism that can regulate vascular remodeling in the tumor microenvironment by directional transfer between tumor cells and endothelial cells, thereby promoting tumor angiogenesis.

### EMT (epithelial‐mesenchymal transition)

EMT is a morphogenic process that results in the loss of intercellular adhesion, the loss of epithelioid characteristics, and the acquisition of mesenchymal phenotype, which is conducive to the migration and invasiveness of tumor cells [104]. EMT is the first step in cancer distant metastasis [105]. Interestingly, recent studies have shown that exosomes are involved in the EMT process [106, 107]. Some studies have shown that CAF mediated HCC tumor progression may be related to the loss of anti-tumor exo-miR-320a in CAF exosomes [68]. It can induce EMT and upregulate the expression of CDK2 and MMP2, thus promoting cell proliferation and metastasis. Increasing the release of stromal cell-derived exo-miR-320a may be a potential therapeutic option for HCC. These results suggest that tumor-derived exogenous microRNAs are important mediators of EMT and can transform tumor cells into more aggressive phenotypes.

### Drug resistance

Chemotherapeutic drugs such as 5-Fluorouracil, oxaliplatin, and gemcitabine are the conventional treatment for advanced HCC patients, but the therapeutic effect is disappointing[108–110]. Multidrug resistance has become the main obstacle in HCC treatment [111]. Therefore, it is important to understand the mechanism of multidrug resistance and explore new treatment targets to overcome multidrug resistance. Cancer resistance can be regulated by exosomes containing mRNA, microRNA and other ncRNAs. It has been confirmed that exo-miR-32-5p, which is transmitted by the exosome of drug-resistant cells, activates PI3k/Akt pathway, and leads to multiple resistance of HCC through promoting angiogenesis and EMT [58]. Another studies have shown that the down-regulated exo-miR-744 can promote the proliferation of HepG2 cells and inhibit the chemical sensitivity of HepG2 cells to sorafeni [112]. The occurrence of cisplatin resistance is one of the main causes of high mortality in patients with liver cancer [113]. It has been found that exo-miR-199a-3p can restore the cisplatin-sensitivity in HCC, and may play a role in the treatment of cisplatin refractory HCC in the future [113]. Therefore, exocrine microRNA can induce or block the resistance of HCC, which plays an important role in the treatment of liver cancer.

### Immune reaction

As the main mediator of intercellular communication and immune function, the immunological activity of exosomes affects the immune regulation mechanism, including the regulation of antigen presentation, immune activation, immunosuppression, immune surveillance, and intercellular communication [114]. As an inflammation-related tumor, the immunosuppressive microenvironment of HCC can promote immune tolerance and escape through a variety of mechanisms. It has been confirmed that exosomal microRNA is involved in the regulation of various immune responses. The study by Peng-Fei Zhang et al. proved that HCC-derived exosomal circUHRF1 up-regulate the expression of exo-miR-449c-5p target gene TIM-3 in NK cells by degrading miR-449c-5p, thereby promoting immune escape and anti-PD1 immunotherapy resistance in HCC [115]. In another related study, NK92 cells with miR-92b overexpressed by transfection with Hep3B-derived exosomes that overexpressed miR-92b significantly inhibited the expression of CD69 in NK92 cells. In addition, the down-regulation of CD69 in NK92 cells affected its cytotoxic effect on Hep3B cells. These results have also been verified in primary NK cells and mouse lymphoma YAC-1 cells, fully demonstrating that the effect of circulating exosomes on the development of liver cancer is partly through the inhibition of NK cell CD69 by liver cancer-derived exo-miR-92b [116]. The study of Liu et al. proved that the endoplasmic reticulum-stressed HCC cells release exosomes to up-regulate the expression of PD-L1 in macrophages, and then inhibit T cell function through the miR-23a-PTEN-Akt pathway [59]. Therefore, exosomal microRNA is involved in various reactions in the immune process of liver cancer, and further understanding of the biological function of exosomal microRNA in liver cancer immune regulation, especially the molecular mechanism involving the interaction of immune cells, maybe tumor-related immunity. The phenomenon of suppression and therapeutic intervention (including immune recognition during malignancy) provides important insights.

## Clinical applications of exosomal microRNA

Exosome microRNAs can serve as the biomarkers for the diagnosis and prognosis of HCC. Liquid biopsy, as an emerging cancer detection technology, has many characteristics such as less-invasiveness, sensitivity and dynamics compared with traditional tissue biopsy. Exosomal microRNAs may represent interesting liquid biopsy markers in cancer, because they can be loaded into the exosomes of tumor cells at a higher level, have high stability and are not easily affected by the external environment. In the modern medical arena, exosomal microRNAs play a critical role in the screening, diagnosis and prognosis of cancer, especially HCC. As early as 2015, Sohn et al. used fluorescent quantitative PCR to detect the expression levels of serum exosomal microRNAs in patients with chronic hepatitis B, liver cirrhosis and HCC, and found that the serum levels of exo-miR-18a, exo-miR-221, exo-miR-222 and exo-miR-224 in patients with HCC were significantly higher than those of patients with chronic hepatitis B or liver cirrhosis. In addition, the serum levels of exo-miR-101, exo-miR-106b, exo-miR-122 and exo-miR-195 in patients with HCC were lower than those in patients with chronic hepatitis B. This study shows that serum exosomal microRNAs can be used as new biomarkers for the screening and diagnosis of HCC [43]. Some researchers have further sequenced and analyzed the exosomal microRNAs of human liver cancer cells and normal liver epithelial cells, and evaluated the clinical application value of differential microRNAs. It was found that serum exo-miR-10b-5p showed up to 91.1 % sensitivity and 75 % specificity, and the area under the curve reached 0.932 in this study. It is a promising biomarker for early detection of HCC. Kaplan-Meier analysis showed that compared with patients with low serum exo-miR-215-5p expression, patients with serum exo-miR-215-5p overexpression had significantly reduced disease-free survival. At the same time, the expression level of exo-miR-215-5p gradually increases with the development of the tumor stage and can be used as a prognostic biomarker for HCC [117]. Another study has found that compared with patients with liver cirrhosis, the expression levels of exo-miR-21 and exo-miR-96 in HCC patients’ exosomes and plasma were significantly increased, and the expression level of exo-miR-122 was significantly reduced. In different populations, exo-miR-122, exo-miR-21 and exo-miR-96 are much more accurate than plasma microRNAs and AFP levels in the diagnosis of HCC, and are promising biomarkers for early detection of HCC [118]. Furthermore, tumor recurrence is generally one of the main reasons for the higher mortality of HCC. In recent years, some exosomal microRNAs have been identified as recurrence-specific biomarkers, especially in patients with HCC. Compared with patients without recurrence, exo-miR-92b was more expressed in patients with recurrence after surgery, and can be used as an important indicator for predicting the risk of HCC recurrence [116]. It can be seen that exosomal microRNAs have good application prospects as markers for the screening, diagnosis and prognosis monitoring.

Exosomal microRNAs have important clinical application values in the diagnosis and prognosis monitoring of HCC, but there are some limitations at present. The first limitation is the extraction of exosomes. There are many ways to purify exosomes at present, but there are big differences in the quantity and quality of the extracted exosomes. The currently accepted method for extracting exosomes is ultracentrifugation, but this method lacks uniform operating standards and takes a long time. In addition, commercial kits on the market for isolating exosomes are very expensive. Therefore, it is necessary to establish a low-cost and time-saving purification method for exosomes. Secondly, different studies on the same exosomal microRNA may draw different conclusions in terms of HCC diagnosis and prognosis assessment. The difference in conclusions is mainly due to the small number of research subjects included in the study and the large individual differences between the research subjects, which leads to inconsistent test results. Therefore, it is necessary to fully consider the various influencing factors of the research object, and further expand the sample size to detect HCC samples at different stages, so as to improve the reliability of serum exosomal microRNA determination results, and make it truly a stable, accurate and effective biomarker for early screening diagnosis and prognosis monitoring of HCC.

The microRNA in exosomes can play an important role in the progression of HCC, however, it rarely translates into therapeutic applications [119]. Since microRNA has a wide range of roles in proliferation, inflammation, and metabolism, it can be considered a powerful therapeutic opportunity in various cancers. Related studies have shown that microRNA-based therapies have been successfully tested, and this type of molecule is preferentially delivered to the liver [120–122]. Based on the important role of exosomal microRNA in the development of liver cancer, Liang JY et al.’s study showed that nanoparticles loaded with siRNA help to inhibit tumor cell migration and the effect the tumorogenic function of exosomes on normal liver cells [123]. By eliminating the oncogenic microRNA in malicious vesicles, a new tumor treatment method can be found. With the improvement of identification, isolation and purification technology, exosomes will be widely used in the clinical treatment of liver cancer.

## Conclusions

**Fig. 1:**
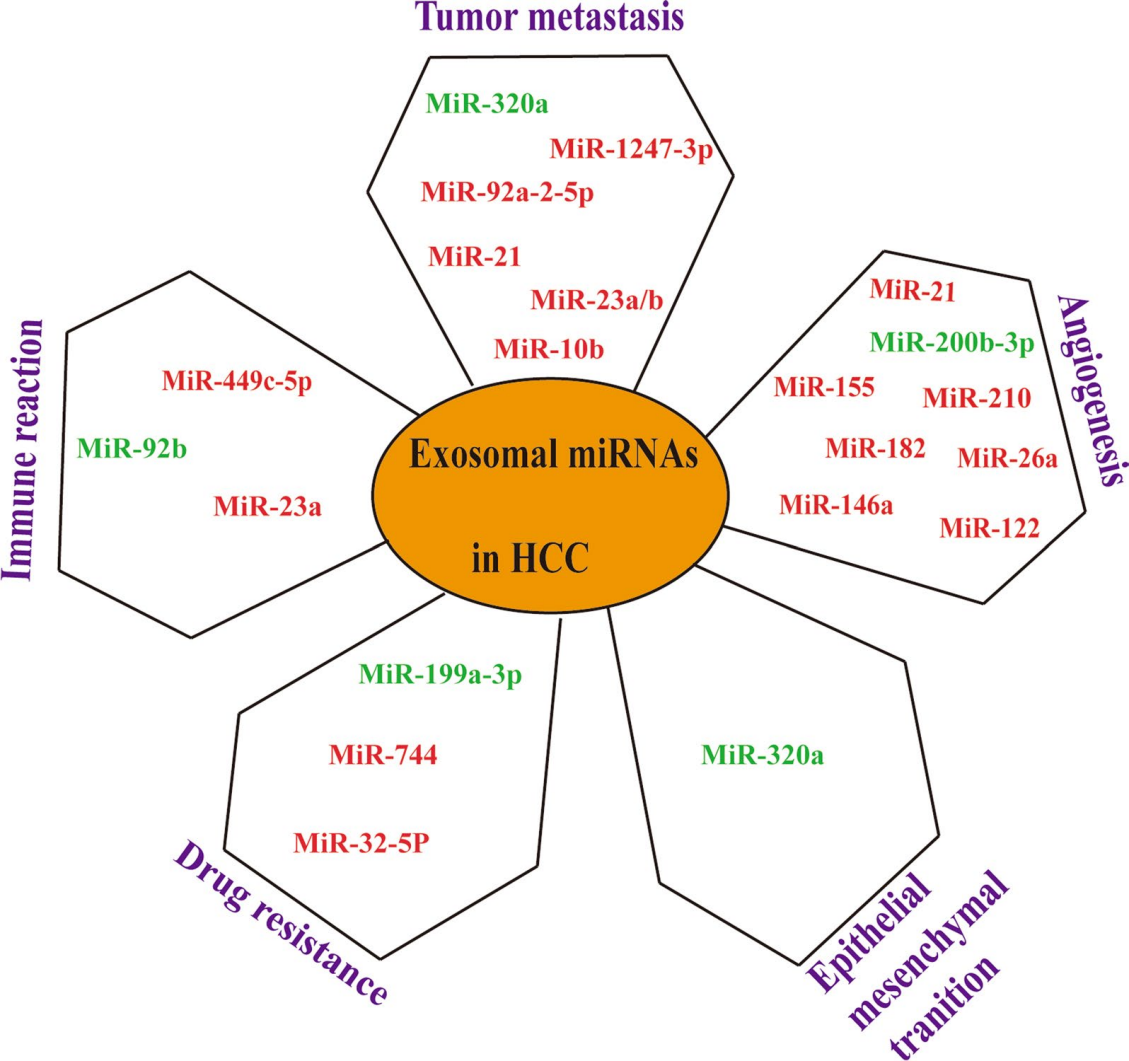
The role of exosomal microRNAs in the HCC microenvironment

Exosomal microRNA participates in the pathological process of many tumors including HCC, and plays an extremely important role in the environmental regulation of the occurrence and development of HCC. Therefore, exosomal microRNA is a promising biomarker for primary lung cancer diagnosis, prediction, prognosis, and real-time monitoring of treatment response. This review summarizes the role and possible mechanisms of exosomes in liver cancer (Table 1; Fig. 1), and shows the potential clinical applications of exosomes in disease detection and treatment (Table 2). However, the detailed mechanism of exosomes in the invasion and metastasis of liver cancer is not fully understood, which hinders its application in the diagnosis and treatment of liver cancer. A deeper understanding of exosomes in liver cancer will provide future diagnosis and treatment of liver cancer. Bring breakthrough changes.

**Table 1:** Exosomal microRNA detected in HCC and clinical relevance

Exosomal MicroRNA	Parental cells	Recipient cells	Expression level in HCC	Target	Potential Mechanism	References
miR-210	HCC cells	Endothelial cells	Upregulated	SMAD4, STAT6	Promote tumor angiogenesis	[42]
miR-744	HepG2 cells		Downregulated	PAX2	Inhibit HepG2 cell proliferation and promotes the chemosensitivity of HepG2 cells to sorafenib	[112]
miR-21, miR-10b	HCC cells		Upregulated in Acidic Microenvironment		Promote cancer cell proliferation and metastasis	[94]
miR-1247-3p	High-metastatic HCC cells	Fibroblasts		B4GALT3	Activate β1-integrin-NF-κB signaling in fibroblasts and promote cancer progression	[91]
miR-451a			Downregulated	LPIN1	Promote apoptosis in HCC and endothelial cells	[78]
miR-23a-3p	ER-stressed HCC cells	Macrophages		PTEN	Regulate PD-L1 expression through the PTEN-PI3K/AKT pathway and help tumor cells escape from antitumor immunity	[59]
miR-32-5p	Multidrug-resistant cell (Bel/5-FU)	Sensitive cell (Bel7402)	Upregulated	PTEN	Activate the PI3K/Akt pathway to further induce multidrug resistance by modulating angiogenesis and EMT	[58]
miR-92b	HCC cells	Natural killer (NK) cells	Upregulated		Promote the downregulation of CD69 and NK cell-mediated cytotoxicity	[116]
miR-320a	Stromal cells	HCC cells	Downregulated	PBX3	Inhibit tumor progression by suppressing the activation of the MAPK pathway	[68]
miR-638	HCC cells	HCC cells, human umbilical vein endothelial cells (HUVECs)	Downregulated	SP1	Repress HCC cell proliferation by decreasing viability and colony formation, and decreased abilities of migration and invasion.	[77]
miR-200b-3p	HCC cells	Endothelial cells	Downregulated		Suppress endothelial ERG expression	[103]

**Table 2:** Exosomal microRNA as diagnostic and prognostic biomarkers in HCC

Exosomal microRNA as biomarkers	Biofluid	Clinical significance	References
exo-miR-215-5p	Serum	Overexpression predicts poor disease-free survival in patients with HCC	[117]
miR-10b-5p	Serum	Potential biomarker for early-stage HCC	[117]
miR-18a, miR-221, miR-222, miR-224, miR-101, miR-106b, miR-122, miR195	Serum	Novel serological biomarkers for HCC	[43]
miR-122, miR-148a and AFP	Serum	Distinguish early HCC from liver cirrhosis	[41]
miR-122	Serum	Distinguish HCC from normal control	[41]
miR-9-3p	Serum	A therapeutic target for HCC	[69]
miR-665	Serum	A novel minimally invasive biomarker for HCC diagnosis and prognosis	[70]
miR-21	Serum	A potential biomarker for HCC diagnosis.	[40]

## Data Availability

Not applicable.

## Abbreviations

HCC: Hepatocellular carcinoma
EMT: Epithelial-mesenchymal transition
RISC: RNA-induced silencing complex
esRNA: Exosome shuttle RNA
AFP: Alpha-fetoprotein
PHC: Primary hepatic carcinoma
PI3K: Phosphatidylinositide 3-kinase
Akt: Serine/threonine kinase
PIP2: Phosphatidylinositol-4,5-bisphosphate
PTEN: Phosphatase and tensin homolog
PD-L1: Programmed Cell Death-Ligand 1
MAPK: Mitogen-activated protein kinase
ERK: Extracellular regulated protein kinases
SAPK: Mitogen-activated protein kinase 9
JNK: C-Jun N-terminal kinase
CDK2: Cyclin-dependent kinase 2
MMP2: Matrix metallopeptidase 2
HUVEC: Human umbilical vein endothelial cell
CAF: Cancer-associated fibroblasts

## References

[1] Villanueva A (2019). Hepatocellular Carcinoma. N Engl J Med.

[2] Craig AJ, von Felden J, Garcia-Lezana T, Sarcognato S, Villanueva A (2020). Tumour evolution in hepatocellular carcinoma. Nat Rev Gastroenterol Hepatol.

[3] Aravalli RN, Steer CJ, Cressman EN (2008). Molecular mechanisms of hepatocellular carcinoma. Hepatology.

[4] Pinzani M, Rosselli M, Zuckermann M (2011). Liver cirrhosis. Best Pract Res Clin Gastroenterol.

[5] El-Serag HB (2012). Epidemiology of viral hepatitis and hepatocellular carcinoma. Gastroenterology.

[6] Schulze K, Nault JC, Villanueva A (2016). Genetic profiling of hepatocellular carcinoma using next-generation sequencing. J Hepatol.

[7] Liu XN, Cui DN, Li YF, Liu YH, Liu G, Liu L (2019). Multiple “Omics” data-based biomarker screening for hepatocellular carcinoma diagnosis. World J Gastroenterol.

[8] Lok AS, Sterling RK, Everhart JE, Wright EC, Hoefs JC, Di Bisceglie AM, Morgan TR, Kim HY, Lee WM, Bonkovsky HL (2010). Des-gamma-carboxy prothrombin and alpha-fetoprotein as biomarkers for the early detection of hepatocellular carcinoma. Gastroenterology.

[9] Marrero JA, Feng Z, Wang Y, Nguyen MH, Befeler AS, Roberts LR, Reddy KR, Harnois D, Llovet JM, Normolle D (2009). Alpha-fetoprotein, des-gamma carboxyprothrombin, and lectin-bound alpha-fetoprotein in early hepatocellular carcinoma. Gastroenterology.

[10] Llovet JM, Bruix J (2003). Systematic review of randomized trials for unresectable hepatocellular carcinoma: Chemoembolization improves survival. Hepatology.

[11] Lo CM, Ngan H, Tso WK, Liu CL, Lam CM, Poon RT, Fan ST, Wong J (2002). Randomized controlled trial of transarterial lipiodol chemoembolization for unresectable hepatocellular carcinoma. Hepatology.

[12] Llovet JM, Real MI, Montaña X, Planas R, Coll S, Aponte J, Ayuso C, Sala M, Muchart J, Solà R (2002). Arterial embolisation or chemoembolisation versus symptomatic treatment in patients with unresectable hepatocellular carcinoma: a randomised controlled trial. Lancet.

[13] Peng Z, Chen S, Wei M, Lin M, Jiang C, Mei J, Li B, Wang Y, Li J, Xie X (2018). Advanced Recurrent Hepatocellular Carcinoma: Treatment with Sorafenib Alone or in Combination with Transarterial Chemoembolization and Radiofrequency Ablation. Radiology.

[14] Cheng AL, Kang YK, Chen Z, Tsao CJ, Qin S, Kim JS, Luo R, Feng J, Ye S, Yang TS (2009). Efficacy and safety of sorafenib in patients in the Asia-Pacific region with advanced hepatocellular carcinoma: a phase III randomised, double-blind, placebo-controlled trial. Lancet Oncol.

[15] Kudo M, Finn RS, Qin S, Han KH, Ikeda K, Piscaglia F, Baron A, Park JW, Han G, Jassem J (2018). Lenvatinib versus sorafenib in first-line treatment of patients with unresectable hepatocellular carcinoma: a randomised phase 3 non-inferiority trial. Lancet.

[16] Bruix J, Qin S, Merle P, Granito A, Huang YH, Bodoky G, Pracht M, Yokosuka O, Rosmorduc O, Breder V (2017). Regorafenib for patients with hepatocellular carcinoma who progressed on sorafenib treatment (RESORCE): a randomised, double-blind, placebo-controlled, phase 3 trial. Lancet.

[17] Abudoureyimu M, Zhou H, Zhi Y, Wang T, Feng B, Wang R, Chu X (2019). Recent progress in the emerging role of exosome in hepatocellular carcinoma. Cell Prolif.

[18] Théry C, Zitvogel L, Amigorena S (2002). Exosomes: composition, biogenesis and function. Nat Rev Immunol.

[19] Sato-Kuwabara Y, Melo SA, Soares FA, Calin GA (2015). The fusion of two worlds: non-coding RNAs and extracellular vesicles–diagnostic and therapeutic implications (Review). Int J Oncol.

[20] Cho JA, Park H, Lim EH, Lee KW (2012). Exosomes from breast cancer cells can convert adipose tissue-derived mesenchymal stem cells into myofibroblast-like cells. Int J Oncol.

[21] Tauro BJ, Mathias RA, Greening DW, Gopal SK, Ji H, Kapp EA, Coleman BM, Hill AF, Kusebauch U, Hallows JL (2013). Oncogenic H-ras reprograms Madin-Darby canine kidney (MDCK) cell-derived exosomal proteins following epithelial-mesenchymal transition. Mol Cell Proteomics.

[22] Kahlert C, Kalluri R (2013). Exosomes in tumor microenvironment influence cancer progression and metastasis. J Mol Med (Berl).

[23] Zhou S, Abdouh M, Arena V, Arena M, Arena GO (2017). Reprogramming Malignant Cancer Cells toward a Benign Phenotype following Exposure to Human Embryonic Stem Cell Microenvironment. PLoS One.

[24] Ruvkun G (2006). Clarifications on miRNA and cancer. Science.

[25] Chen SY, Ma DN, Chen QD, Zhang JJ, Tian YR, Wang ZC, Cai H, Lin Y, Sun HC (2017). MicroRNA-200a inhibits cell growth and metastasis by targeting Foxa2 in hepatocellular carcinoma. J Cancer.

[26] Png KJ, Halberg N, Yoshida M, Tavazoie SF (2011). A microRNA regulon that mediates endothelial recruitment and metastasis by cancer cells. Nature.

[27] Gee HE, Camps C, Buffa FM, Colella S, Sheldon H, Gleadle JM, Ragoussis J, Harris AL (2008). MicroRNA-10b and breast cancer metastasis. Nature.

[28] Tay Y, Zhang J, Thomson AM, Lim B, Rigoutsos I (2008). MicroRNAs to Nanog, Oct4 and Sox2 coding regions modulate embryonic stem cell differentiation. Nature.

[29] Kota J, Chivukula RR, O’Donnell KA, Wentzel EA, Montgomery CL, Hwang HW, Chang TC, Vivekanandan P, Torbenson M, Clark KR (2009). Therapeutic microRNA delivery suppresses tumorigenesis in a murine liver cancer model. Cell.

[30] Lotvall J, Valadi H (2007). Cell to cell signalling via exosomes through esRNA. Cell Adh Migr.

[31] Kosaka N, Takeshita F, Yoshioka Y, Hagiwara K, Katsuda T, Ono M, Ochiya T (2013). Exosomal tumor-suppressive microRNAs as novel cancer therapy: “exocure” is another choice for cancer treatment. Adv Drug Deliv Rev.

[32] Sun Z, Shi K, Yang S, Liu J, Zhou Q, Wang G, Song J, Li Z, Zhang Z, Yuan W (2018). Effect of exosomal miRNA on cancer biology and clinical applications. Mol Cancer.

[33] Li C, Xu X (2019). Biological functions and clinical applications of exosomal non-coding RNAs in hepatocellular carcinoma. Cell Mol Life Sci.

[34] Szabo G, Momen-Heravi F (2017). Extracellular vesicles in liver disease and potential as biomarkers and therapeutic targets. Nat Rev Gastroenterol Hepatol.

[35] Liu C, Yang Y, Wu Y (2018). Recent advances in exosomal protein detection via liquid biopsy biosensors for cancer screening, diagnosis, and prognosis. Aaps J.

[36] Fais S, Logozzi M, Lugini L, Federici C, Azzarito T, Zarovni N, Chiesi A (2013). Exosomes: the ideal nanovectors for biodelivery. Biol Chem.

[37] Sasaki R, Kanda T, Yokosuka O, Kato N, Matsuoka S, Moriyama M (2019). Exosomes and Hepatocellular Carcinoma: from bench to bedside. Int J Mol Sci.

[38] Chen R, Xu X, Tao Y, Qian Z, Yu Y (2019). Exosomes in hepatocellular carcinoma: a new horizon. Cell Commun Signal.

[39] Li X, Li C, Zhang L, Wu M, Cao K, Jiang F, Chen D, Li N, Li W (2020). The significance of exosomes in the development and treatment of hepatocellular carcinoma. Mol Cancer.

[40] Wang H, Hou L, Li A, Duan Y, Gao H, Song X (2014). Expression of serum exosomal microRNA-21 in human hepatocellular carcinoma. Biomed Res Int.

[41] Wang Y, Zhang C, Zhang P, Guo G, Jiang T, Zhao X, Jiang J, Huang X, Tong H, Tian Y (2018). Serum exosomal microRNAs combined with alpha-fetoprotein as diagnostic markers of hepatocellular carcinoma. Cancer Med.

[42] Lin XJ, Fang JH, Yang XJ, Zhang C, Yuan Y, Zheng L, Zhuang SM (2018). Hepatocellular carcinoma cell-secreted exosomal microRNA-210 promotes angiogenesis in vitro and in vivo. Mol Ther Nucleic Acids.

[43] Sohn W, Kim J, Kang SH, Yang SR, Cho JY, Cho HC, Shim SG, Paik YH (2015). Serum exosomal microRNAs as novel biomarkers for hepatocellular carcinoma. Exp Mol Med.

[44] Goldsmith JR, Fayngerts S, Chen YH (2017). Regulation of inflammation and tumorigenesis by the TIPE family of phospholipid transfer proteins. Cell Mol Immunol.

[45] Cantley LC (2002). The phosphoinositide 3-kinase pathway. Science.

[46] Simpson L, Parsons R (2001). PTEN: life as a tumor suppressor. Exp Cell Res.

[47] Song G, Ouyang G, Bao S (2005). The activation of Akt/PKB signaling pathway and cell survival. J Cell Mol Med.

[48] Franke TF, Kaplan DR, Cantley LC (1997). PI3K: downstream AKTion blocks apoptosis. Cell.

[49] Burgering BM, Coffer PJ (1995). Protein kinase B (c-Akt) in phosphatidylinositol-3-OH kinase signal transduction. Nature.

[50] Franke TF, Yang SI, Chan TO, Datta K, Kazlauskas A, Morrison DK, Kaplan DR, Tsichlis PN (1995). The protein kinase encoded by the Akt proto-oncogene is a target of the PDGF-activated phosphatidylinositol 3-kinase. Cell.

[51] Hajduch E, Litherland GJ, Hundal HS (2001). Protein kinase B (PKB/Akt)--a key regulator of glucose transport?. FEBS Lett.

[52] Gesbert F, Sellers WR, Signoretti S, Loda M, Griffin JD (2000). BCR/ABL regulates expression of the cyclin-dependent kinase inhibitor p27Kip1 through the phosphatidylinositol 3-Kinase/AKT pathway. J Biol Chem.

[53] Zhou BP, Liao Y, Xia W, Spohn B, Lee MH, Hung MC (2001). Cytoplasmic localization of p21Cip1/WAF1 by Akt-induced phosphorylation in HER-2/neu-overexpressing cells. Nat Cell Biol.

[54] Yu HG, Ai YW, Yu LL, Zhou XD, Liu J, Li JH, Xu XM, Liu S, Chen J, Liu F (2008). Phosphoinositide 3-kinase/Akt pathway plays an important role in chemoresistance of gastric cancer cells against etoposide and doxorubicin induced cell death. Int J Cancer.

[55] Zhang K, Chen J, Chen D, Huang J, Feng B, Han S, Chen Y, Song H, De W, Zhu Z (2014). Aurora-A promotes chemoresistance in hepatocelluar carcinoma by targeting NF-kappaB/microRNA-21/PTEN signaling pathway. Oncotarget.

[56] Zhou W, Fu XQ, Zhang LL, Zhang J, Huang X, Lu XH, Shen L, Liu BN, Liu J, Luo HS (2013). The AKT1/NF-kappaB/Notch1/PTEN axis has an important role in chemoresistance of gastric cancer cells. Cell Death Dis.

[57] Zhang L, Wang W, Li X, He S, Yao J, Wang X, Zhang D, Sun X (2016). MicroRNA-155 promotes tumor growth of human hepatocellular carcinoma by targeting ARID2. Int J Oncol.

[58] Fu X, Liu M, Qu S, Ma J, Zhang Y, Shi T, Wen H, Yang Y, Wang S, Wang J (2018). Exosomal microRNA-32-5p induces multidrug resistance in hepatocellular carcinoma via the PI3K/Akt pathway. J Exp Clin Cancer Res.

[59] Liu J, Fan L, Yu H, Zhang J, He Y, Feng D, Wang F, Li X, Liu Q, Li Y, Guo Z, Gao B, Wei W, Wang H, Sun G. Endoplasmic reticulum stress causes liver cancer cells to release exosomal miR-23a-3p and up-regulate programmed death ligand 1 expression in macrophages. Hepatology. 2019. 10.1002/hep.30607 PMC6597282 30854665

[60] Chen H, Wong CC, Liu D, Go MYY, Wu B, Peng S, Kuang M, Wong N, Yu J (2019). APLN promotes hepatocellular carcinoma through activating PI3K/Akt pathway and is a druggable target. Theranostics.

[61] Nunnery SE, Mayer IA (2019). Management of toxicity to isoform α-specific PI3K inhibitors. Ann Oncol.

[62] Turjanski AG, Vaqué JP, Gutkind JS (2007). MAP kinases and the control of nuclear events. Oncogene.

[63] Kim EK, Choi EJ (2015). Compromised MAPK signaling in human diseases: an update. Arch Toxicol.

[64] Roux PP, Blenis J (2004). ERK and p38 MAPK-activated protein kinases: a family of protein kinases with diverse biological functions. Microbiol Mol Biol Rev.

[65] Baccarini M (2005). Second nature: biological functions of the Raf-1 “kinase”. FEBS Lett.

[66] Meloche S, Pouysségur J (2007). The ERK1/2 mitogen-activated protein kinase pathway as a master regulator of the G1- to S-phase transition. Oncogene.

[67] Roberts PJ, Der CJ (2007). Targeting the Raf-MEK-ERK mitogen-activated protein kinase cascade for the treatment of cancer. Oncogene.

[68] Zhang Z, Li X, Sun W, Yue S, Yang J, Li J, Ma B, Wang J, Yang X, Pu M (2017). Loss of exosomal miR-320a from cancer-associated fibroblasts contributes to HCC proliferation and metastasis. Cancer Lett.

[69] Tang J, Li Y, Liu K, Zhu Q, Yang WH, Xiong LK, Guo DL (2018). Exosomal miR-9-3p suppresses HBGF-5 expression and is a functional biomarker in hepatocellular carcinoma. Minerva Med.

[70] Qu Z, Wu J, Wu J, Ji A, Qiang G, Jiang Y, Jiang C, Ding Y (2017). Exosomal miR-665 as a novel minimally invasive biomarker for hepatocellular carcinoma diagnosis and prognosis. Oncotarget.

[71] Xiong L, Zhen S, Yu Q, Gong Z (2017). HCV-E2 inhibits hepatocellular carcinoma metastasis by stimulating mast cells to secrete exosomal shuttle microRNAs. Oncol Lett.

[72] Kumar B, Sinclair J, Khandrika L, Koul S, Wilson S, Koul HK (2009). Differential effects of MAPKs signaling on the growth of invasive bladder cancer cells. Int J Oncol.

[73] Pistritto G, Trisciuoglio D, Ceci C, Garufi A, D’Orazi G (2016). Apoptosis as anticancer mechanism: function and dysfunction of its modulators and targeted therapeutic strategies. Aging.

[74] Yaacoub K, Pedeux R, Tarte K, Guillaudeux T (2016). Role of the tumor microenvironment in regulating apoptosis and cancer progression. Cancer Lett.

[75] Haikala HM, Anttila JM, Marques E, Raatikainen T, Ilander M, Hakanen H, Ala-Hongisto H, Savelius M, Balboa D, Von Eyss B (2019). Pharmacological reactivation of MYC-dependent apoptosis induces susceptibility to anti-PD-1 immunotherapy. Nat Commun.

[76] Alzahrani FA, El-Magd MA, Abdelfattah-Hassan A, Saleh AA, Saadeldin IM, El-Shetry ES, Badawy AA, Alkarim S (2018). Potential effect of exosomes derived from cancer stem cells and MSCs on progression of DEN-induced HCC in rats. Stem Cells Int.

[77] Yang J, Li B, Zhao S, Du H, Du Y (2020). Exosomal miR-638 Inhibits Hepatocellular Carcinoma Progression by Targeting SP1. Onco Targets Ther.

[78] Zhao S, Li J, Zhang G, Wang Q, Wu C, Zhang Q, Wang H, Sun P, Xiang R, Yang S (2019). Exosomal miR-451a Functions as a Tumor Suppressor in Hepatocellular Carcinoma by Targeting LPIN1. Cell Physiol Biochem.

[79] Hassan M, Watari H, AbuAlmaaty A, Ohba Y, Sakuragi N (2014). Apoptosis and molecular targeting therapy in cancer. BioMed Res Int.

[80] Tsuruo T, Naito M, Tomida A, Fujita N, Mashima T, Sakamoto H, Haga N (2003). Molecular targeting therapy of cancer: drug resistance, apoptosis and survival signal. Cancer Sci.

[81] Xiao F, Xiao S, Xue M: miR-139 controls viability of ovarian cancer cells through apoptosis induction and exosome shedding inhibition by targeting ATP7A. *OncoTargets Ther *2019; **12**:10727–10737. 10.2147/OTT.S221236 PMC6904246 31839712

[82] Wang M, Yu F, Li P. Circular RNAs: Characteristics, function and clinical significance in hepatocellular carcinoma. *Cancers* 2018; 10: 8. 10.3390/cancers10080258 PMC6116001 30072625

[83] van Zijl F, Krupitza G, Mikulits W (2011). Initial steps of metastasis: cell invasion and endothelial transmigration. Mutat Res.

[84] Zhou Y, Yamamoto Y, Takeshita F, Yamamoto T, Xiao Z, Ochiya T. Delivery of miR-424-5p via Extracellular vesicles promotes the apoptosis of MDA-MB-231 TNBC cells in the tumor microenvironment. *Int J Mol Sci *2021, 22:2. 10.3390/ijms22020844 PMC7831022 33467725

[85] He M, Qin H, Poon TC, Sze SC, Ding X, Co NN, Ngai SM, Chan TF, Wong N (2015). Hepatocellular carcinoma-derived exosomes promote motility of immortalized hepatocyte through transfer of oncogenic proteins and RNAs. Carcinogenesis.

[86] Liu WH, Ren LN, Wang X, Wang T, Zhang N, Gao Y, Luo H, Navarro-Alvarez N, Tang LJ (2015). Combination of exosomes and circulating microRNAs may serve as a promising tumor marker complementary to alpha-fetoprotein for early-stage hepatocellular carcinoma diagnosis in rats. J Cancer Res Clin Oncol.

[87] Zhang H, Deng T, Liu R, Bai M, Zhou L, Wang X, Li S, Wang X, Yang H, Li J (2017). Exosome-delivered EGFR regulates liver microenvironment to promote gastric cancer liver metastasis. Nat Commun.

[88] Yu Z, Zhao S, Ren L, Wang L, Chen Z, Hoffman RM, Zhou J (2017). Pancreatic cancer-derived exosomes promote tumor metastasis and liver pre-metastatic niche formation. Oncotarget.

[89] Plebanek MP, Angeloni NL, Vinokour E, Li J, Henkin A, Martinez-Marin D, Filleur S, Bhowmick R, Henkin J, Miller SD (2017). Pre-metastatic cancer exosomes induce immune surveillance by patrolling monocytes at the metastatic niche. Nat Commun.

[90] Li L, Li C, Wang S, Wang Z, Jiang J, Wang W, Li X, Chen J, Liu K, Li C (2016). Exosomes Derived from Hypoxic Oral Squamous Cell Carcinoma Cells Deliver miR-21 to Normoxic Cells to Elicit a Prometastatic Phenotype. Cancer Res.

[91] Fang T, Lv H, Lv G, Li T, Wang C, Han Q, Yu L, Su B, Guo L, Huang S (2018). Tumor-derived exosomal miR-1247-3p induces cancer-associated fibroblast activation to foster lung metastasis of liver cancer. Nat Commun.

[92] Liu G, Ouyang X, Sun Y, Xiao Y, You B, Gao Y, Yeh S, Li Y, Chang C. The miR-92a-2-5p in exosomes from macrophages increases liver cancer cells invasion via altering the AR/PHLPP/p-AKT/β-catenin signaling. Cell Death Differ 2020. 10.1038/s41418-020-0575-3 PMC7853149 32587378

[93] Liu Y, Tan J, Ou S, Chen J, Chen L (2019). Adipose-derived exosomes deliver miR-23a/b to regulate tumor growth in hepatocellular cancer by targeting the VHL/HIF axis. J Physiol Biochem.

[94] Tian XP, Wang CY, Jin XH, Li M, Wang FW, Huang WJ, Yun JP, Xu RH, Cai QQ, Xie D (2019). Acidic Microenvironment Up-Regulates Exosomal miR-21 and miR-10b in Early-Stage Hepatocellular Carcinoma to Promote Cancer Cell Proliferation and Metastasis. Theranostics.

[95] Carmeliet P, Jain RK (2000). Angiogenesis in cancer and other diseases. Nature.

[96] Folkman J (2002). Role of angiogenesis in tumor growth and metastasis. Seminars in oncology.

[97] Huang CS, Yu W, Cui H, Wang YJ, Zhang L, Han F, Huang T (2015). Increased expression of miR-21 predicts poor prognosis in patients with hepatocellular carcinoma. Int J Clin Exp Pathol.

[98] Yang X, Zhang XF, Lu X, Jia HL, Liang L, Dong QZ, Ye QH, Qin LX (2014). MicroRNA-26a suppresses angiogenesis in human hepatocellular carcinoma by targeting hepatocyte growth factor-cMet pathway. Hepatology.

[99] Xu Q, Zhang M, Tu J, Pang L, Cai W, Liu X (2015). MicroRNA-122 affects cell aggressiveness and apoptosis by targeting PKM2 in human hepatocellular carcinoma. Oncol Rep.

[100] Zhu K, Pan Q, Zhang X, Kong LQ, Fan J, Dai Z, Wang L, Yang XR, Hu J, Wan JL (2013). MiR-146a enhances angiogenic activity of endothelial cells in hepatocellular carcinoma by promoting PDGFRA expression. Carcinogenesis.

[101] Guan C, Yang F, He X, Li T, Yang Q, He H, Xu M (2016). Clinical significance of microRNA-155 expression in hepatocellular carcinoma. Oncol Lett.

[102] Chen L, Chu F, Cao Y, Shao J, Wang F (2015). Serum miR-182 and miR-331-3p as diagnostic and prognostic markers in patients with hepatocellular carcinoma. Tumour Biol.

[103] Moh-Moh-Aung A, Fujisawa M, Ito S, Katayama H, Ohara T, Ota Y, Yoshimura T, Matsukawa A (2020). Decreased miR-200b-3p in cancer cells leads to angiogenesis in HCC by enhancing endothelial ERG expression. Sci Rep.

[104] Greening DW, Gopal SK, Mathias RA, Liu L, Sheng J, Zhu HJ, Simpson RJ (2015). Emerging roles of exosomes during epithelial-mesenchymal transition and cancer progression. Semin Cell Dev Biol.

[105] Markopoulos GS, Roupakia E, Tokamani M, Chavdoula E, Hatziapostolou M, Polytarchou C, Marcu KB, Papavassiliou AG, Sandaltzopoulos R, Kolettas E (2017). A step-by-step microRNA guide to cancer development and metastasis. Cell Oncol (Dordr).

[106] Blackwell RH, Foreman KE, Gupta GN. The role of cancer-derived exosomes in tumorigenicity & epithelial-to-mesenchymal transition. Cancers*.* 2017; 9:8. 10.3390/cancers9080105 PMC5575608 28796150

[107] Syn N, Wang L, Sethi G, Thiery JP, Goh BC (2016). Exosome-Mediated Metastasis: From Epithelial-Mesenchymal Transition to Escape from Immunosurveillance. Trends Pharmacol Sci.

[108] Shen YC, Lin ZZ, Hsu CH, Hsu C, Shao YY, Cheng AL (2013). Clinical trials in hepatocellular carcinoma: an update. Liver Cancer.

[109] Kalyan A, Nimeiri H, Kulik L (2015). Systemic therapy of hepatocellular carcinoma: current and promising. Clin Liver Dis.

[110] Llovet JM, Villanueva A, Lachenmayer A, Finn RS (2015). Advances in targeted therapies for hepatocellular carcinoma in the genomic era. Nat Rev Clin Oncol.

[111] Llovet JM, Hernandez-Gea V (2014). Hepatocellular carcinoma: reasons for phase III failure and novel perspectives on trial design. Clin Cancer Res.

[112] Wang G, Zhao W, Wang H, Qiu G, Jiang Z, Wei G, Li X (2019). Exosomal MiR-744 Inhibits Proliferation and Sorafenib Chemoresistance in Hepatocellular Carcinoma by Targeting PAX2. Med Sci Monit.

[113] Zhang K, Shao CX, Zhu JD, Lv XL, Tu CY, Jiang C, Shang MJ. Exosomes function as nanoparticles to transfer miR-199a-3p to reverse chemoresistance to cisplatin in hepatocellular carcinoma. Biosci Rep 2020. 10.1042/BSR20194026 PMC7341182 32463473

[114] Greening DW, Gopal SK, Xu R, Simpson RJ, Chen W (2015). Exosomes and their roles in immune regulation and cancer. Semin Cell Dev Biol.

[115] Zhang PF, Gao C, Huang XY, Lu JC, Guo XJ, Shi GM, Cai JB, Ke AW (2020). Cancer cell-derived exosomal circUHRF1 induces natural killer cell exhaustion and may cause resistance to anti-PD1 therapy in hepatocellular carcinoma. Mol Cancer.

[116] Nakano T, Chen IH, Wang CC, Chen PJ, Tseng HP, Huang KT, Hu TH, Li LC, Goto S, Cheng YF (2019). Circulating exosomal miR-92b: Its role for cancer immunoediting and clinical value for prediction of posttransplant hepatocellular carcinoma recurrence. Am J Transplant.

[117] Cho HJ, Eun JW, Baek GO, Seo CW, Ahn HR, Kim SS, Cho SW, Cheong JY (2020). Serum exosomal MicroRNA, miR-10b-5p, as a Potential diagnostic biomarker for early-stage hepatocellular carcinoma. J Clin Med.

[118] Wang S, Yang Y, Sun L, Qiao G, Song Y, Liu B (2020). Exosomal MicroRNAs as Liquid Biopsy Biomarkers in Hepatocellular Carcinoma. OncoTargets therapy.

[119] Wang H, Lu Z, Zhao X (2019). Tumorigenesis, diagnosis, and therapeutic potential of exosomes in liver cancer. J Hematol Oncol.

[120] Gougelet A, Sartor C, Bachelot L, Godard C, Marchiol C, Renault G, Tores F, Nitschke P, Cavard C, Terris B (2016). Antitumour activity of an inhibitor of miR-34a in liver cancer with β-catenin-mutations. Gut.

[121] Shibata C, Otsuka M, Kishikawa T, Ohno M, Yoshikawa T, Takata A, Koike K (2015). Diagnostic and therapeutic application of noncoding RNAs for hepatocellular carcinoma. World J Hepatol.

[122] Roberts J, Palma E, Sazani P, Ørum H, Cho M, Kole R (2006). Efficient and persistent splice switching by systemically delivered LNA oligonucleotides in mice. Mol Ther.

[123] Liang J, Zhang X, He S, Miao Y, Wu N, Li J, Gan Y (2018). Sphk2 RNAi nanoparticles suppress tumor growth via downregulating cancer cell derived exosomal microRNA. J Control Release.

